# Factors contributing to low uptake and renewal of health insurance: a qualitative study in Ghana

**DOI:** 10.1186/s41256-016-0018-3

**Published:** 2016-11-22

**Authors:** Ama Pokuaa Fenny, Anthony Kusi, Daniel K. Arhinful, Felix Ankoma Asante

**Affiliations:** 1 Economics Division, Institute of Statistical, Social and Economic Research (ISSER), University of Ghana, PO Box LG 74, Legon, LG74 Accra, Ghana; 2 Department of Epidemiology, Noguchi Memorial Institute for Medical Research, University of Ghana, Accra, Ghana

**Keywords:** National Health Insurance, Sociocultural, Barriers to health care, Qualitative study

## Abstract

**Background:**

The effort to expand access to healthcare and reduce health inequalities in many low income countries have meant that many have adopted different levels of social health protection mechanisms. Ghana introduced a National Health Insurance Scheme (NHIS) in 2005 with the aim of removing previous barriers created by the user fees financing system. Although the NHIS has made health accessible to some category of people, the majority of Ghanaians (60 %) are not enroled on the scheme. Earlier studies have looked at various factors that account for this low uptake. However, we recognise that this qualitative study will nuance the depth of these barriers to enrolment.

**Methods:**

Minimally structured, qualitative interviews were conducted with key stakeholders at the district, regional and national levels. Focus group discussions were also undertaken at the community level. Using an inductive and content analytic approach, the transcripts were analyzed to identify and define categories that explain low uptake of health insurance.

**Results:**

The results are presented under two broad themes: sociocultural and systemic factors. Sociocultural factors identified were 1) vulnerability within certain groups such as the aged and the disabled groups which impeded access to the NHIS 2) cultural and religious norms which discouraged enrolment into the scheme. System-wide factors were 1) inadequate distribution of social infrastructure such as healthcare facilities, 2) weak administrative processes within the NHIS, and 3) poor quality of care.

**Conclusions:**

Mapping the interplay of these dynamic relations between the NHIS, its clients and service providers, the study identifies critical factors at the policy-making level, service provider level, and client level (reflective in household and community level institutional arrangements) that affect enrolment in the scheme. Our findings inform a number of potential reforms in the area of distribution of health resources and cost containment to expand coverage, increase choices and meeting the needs of the end user.

## Background

In 2005, the World Health Assembly resolution 58.33 called for a global situation in which “everyone should be able to access health services and not be subject to financial hardship in doing so” [1]. This call followed from the recognition by the WHO in the year 2000 that prepayment of healthcare services was the best form of revenue collection to guarantee access to health care especially for the poor. Indeed, recent global statistics show that out-of-pocket payment for health care is quite high in Lower-Middle-Income Countries (LMICs), a situation which could impede access to health care. While out-of-pocket expenditure as percentage of private expenditure on health is 38.5 % in high income countries, the figure is 86.7 % for lower middle income countries and 77.6 % in low income countries [2]. However, a major challenge toward the goal of achieving universal health coverage is the low enrolment and retention rates in social health insurance schemes [3, 4].

Ghana’s response to the call for universal coverage was the introduction of a National Health Insurance Scheme (NHIS) in 2005 with the aim of removing previous barriers created by the user fees financing system and in an effort to expand access to healthcare and reduce health inequalities. Despite this, there is still striking evidence of rural–urban disparities in access to health care services, inequitable distribution of health workers and gender gaps in access [5, 6]. It has become quite obvious that removing financial barriers alone does not necessarily guarantee equitable access to health care. There are other barriers to accessing health care which can explain the low rates of enrolment in the scheme. The scheme covers about 40% of the total population.

Several factors have been attributed to the low enrolment and retention rates in many of these prepayments schemes especially in developing countries. The common ones include inconsistent or incomplete policies regarding eligibility, preferential enrolment of formal and civil sector workers, inability to identify and enrol vulnerable population, lack of portability, lack of understanding of the insurance concept, affordability, lack of trust in insurers and unfavorable timing of the premium payment [7–9]. In Ghana, past studies have identified these factors to include: unavailability of health facilities, travel cost to health facilities, perceived quality of service, travel cost to NHIS registration centres and socio-cultural factors. Patients enroled in the scheme, complain of poor quality of health services provided at the health facilities which include long waiting times, bad attitude by health facility staff, and drug shortages [10, 11].

The study aims to use an inductive approach to category construction and a content analysis framework to better understand what might be some of the major reasons why individuals did not enrol in the scheme. There is vast literature on enrolment seeking behaviour and the NHIS using quantitative methods [12–15] but very few using qualitative methods. The results will offer a more in-depth understanding of what these barriers to enrolment are and help provide the basis for practical solutions to remove them.

### Brief overview of the NHIS

The NHIS is currently financed by pooled contributions from a value-added tax of 2.5 % earmarked for national health insurance which forms about 66 % of NHIS revenues; interest earned on National Health Insurance Fund (NHIF) reserves; 2.5 % of social security contributions from formal sector workers; insurance premiums from informal sector workers which accounts for 4 % of NHIS revenues. The scheme requires individuals to renew their enrolment through annual registration at district NHIS offices or other designated points. This ensures an uninterrupted access to health care.

Membership in the NHIS is mandatory for all residents of the country as stipulated by the National Health Insurance Act of 2012 (Act 852). The scheme however has an exemption policy in place to ensure that the very poor and vulnerable groups in the society have access to free healthcare. The premium exempt groups include the very poor (i.e. indigents in the community identified by community leaders and social workers and referred to the NHIS), children under the age of 18 years and the elderly (70 years and above). Pregnant women are also exempted from paying premiums. Premiums varied from district to district but could range between a minimum of 7.2 Ghana Cedis (US$4.8) and a maximum of 48.0 Ghana Cedis (US$32.0) [14]. All individuals in a particular district however pay the same amount as determined by that district, irrespective of one’s socio-economic status.

Health services are provided to scheme members through over 3500 public and private health facilities accredited by the NHIA. Facilities are required to meet quality stands with regards to a specified number of qualified health personnel, availability and quality of utilities such as regular supply of water and electricity among others. It also stipulates post-accreditation monitoring to ensure that medical procedure and the administration of drugs are appropriate and comply with the accepted medical practice and ethics. The scheme’s emphasis on quality of care provided at facilities is meant to encourage the insured members to remain insured and also draw in new members. Valid NHIS cardholders can access care at National Health Insurance Authority (NHIA)-accredited public, mission, and private for-profit health facilities.

## Methods

### Study design

This study is a descriptive design with the qualitative approach of content analysis. Qualitative data collection was undertaken in two parts. These were focus group discussions (FGDs) and key informant interviews via semi-structured, face-to-face interviews. The purpose of FGDs was to know the perceptions and views of individuals on the performance of the NHIS and to identify the barriers they face with the scheme service providers. Each of the potential target groups was stratified by sex (male/female) as shown in Table 1.

**Table 1:** Target groups for focused group discussions

Targeted population	Number of groups
Never insured	10
Previously insured	10
Registered but yet to received ID card	10
Currently insured (Valid card holders)	10
Total	40

For the key informant interviews, the initial set of stakeholders was identified by literature review and grouped into broad categories such as the Ghana Health Service (GHS), Non-Governmental Organisations (NGOs), Civil Society Organisations (CSOs), health providers and NHIS managers. Key informants from each of these broad categories were identified. These included district health directors, medical superintendents, hospital administrators (both public and private), district social welfare officers, and district managers of the NHIS as well as opinion and traditional leaders of the studied communities (Table 2). Opinion leaders are usually members in the local communities who advocate for social justice often calling for the provision of social amenities such us hospitals, schools ad roads.

**Table 2:** Category of key informants

Category	Number
Directors of health services	5
Scheme managers	5
Medical superintendents	5
Hospital administrators-public	4
Hospital administrators-private	4
Social welfare officers	5
Medical assistants/Health workers	5
Community leaders/opinion leaders	12
Total	46

### Study setting

The qualitative study took place in five selected districts across the three ecological zones of Ghana, namely the coastal, forest and savannah. These selected districts presented high risk of exclusion, for example rural poor districts so as to have both a significant sample of excluded individuals, but also still having the voluntarily excluded populations. The Abura-Asebu-Kwamamkese was selected in the coastal zone. The district is bordered by the Cape Coast Municipality in the Central region and has a population of 90,093. The Health Services of the district are organised around a full-fledged District Hospital and 69 community-based outreach clinics and 32 Traditional Birth Attendant outposts. Kwaebibirem district was selected in the coastal agro-ecological zone. The district is located in the South Western part of the Eastern region and has a population of 205,932. Kwaebibirem has 33 health facilities, comprising of district hospitals, health centres, clinics and Community-based Health Planning and Services (CHPS) compounds. The third was the Ejisu-Juaben Municipal is one of the 27 administrative and political districts in the Ashanti Region within the forest zone with a population of about 144,272. The district has 1 district hospital and other health facilities like clinics, health posts and maternity homes. Also in the forest agro-ecological zone, the Asutifi district was selected. It is located in the southeast of the Brong Ahafo region with a population of 108,993. The district has 16 health facilities serving six-sub districts also comprising one district hospital, health centres, clinics and CHPS compounds. The last district is the Savelugu/Nanton in the savanna region which shares boundaries with the Tamale metropolis, the capital city of the Northern region with a population of 118,582. The district has 13 health facilities with the Savelugu District hospital serving as the district hospital. The choice of these districts was informed by their level of development and previous working experience in these districts by the Institute of Statistical, Social and Economic Research (ISSER) team. They are all rural districts and relatively underdeveloped.

### Sampling, recruitment, and enrolment

We used a purposive sampling strategy to guide enrolment, which sought to identify groups of individuals having knowledge and experience with the NHIS in Ghana. Findings from the literature review and initial stakeholder analysis provided the needed information for the formation of the groups. In each of the 5 districts, a total of 8 focus group discussions were conducted bring the overall total of FGDs to 40. Each of the target groups was stratified by sex (male/female). In some districts, the focus groups were mixed (both males and females). Each individual was presented with an informed consent form prior to the discussions which was held in the local language. No remuneration was provided for those who chose to participate. A total of 46 stakeholders were interviewed through key informant interviews in five districts across the country. The process of recruitment, enrolment, interview, and quality review typically spanned 14 days from beginning to completion per each district. This study was completed in one district before moving on to the next. Qualitative data analysis was ongoing throughout the study period, as transcripts were reviewed for content in the field.

### Data collection

Interviews were conducted between May and July 2012 by four researchers including two of the co-authors. The four researchers had familiarity with local customs and cultural norms, and were fluent in the local languages used in this study. These researchers were trained in qualitative methods as well as guided through the principles of research ethics that guide social research such as respect for respondents, beneficence, and justice. Interviews were conducted in either the local languages or English, based on the preference of the participant.

### Ethical considerations

Ethical clearance, review number 019/10-11 was sought and granted from the Institutional Review Board (IRB), of the Noguchi Memorial Institute for Medical Research (NMIMR), University of Ghana before the study was done. Study objectives, benefits, risks and the right to refuse participation and confidentiality of responses were explained to participants. Written informed consent was obtained from each participant.

### Data analysis

The data was analyzed using an inductive approach to category construction and a content analysis framework [16]. Inductive content analysis is a qualitative method of content analysis used to develop theory and identify themes by studying documents, recordings and other printed and verbal material [17]. Themes emerge from the raw data through repeated examination and comparison. In this study, interview transcripts were reviewed for content pertaining to barriers to enrolment by eligible individuals through focused group discussions and key informant interviews. On the basis of this content, a coding system was developed that represented common topics encountered in the transcript review. Codes were sharpened and refined throughout the data analysis period, as new transcripts were analyzed shortly after interview completion. This process continued until new interview data did not alter the definition or scope of the codes. Transcripts were reviewed and entered into NVivo 10, a qualitative software analysis package. The data was coded for analysis using QRS NVivo 10 and exported into a Microsoft word for write up. The coded data were scrutinized, grouped, and re-grouped in an effort to reveal major patterns that might indicate an underlying theme or explanation for barriers to enrolment into the NHIS. From this process, descriptive categories were developed to show the factors which were then characterized with a “name” to describe a basis of explanation for the observed phenomenon. These categories were revised, refined, and validated through data triangulation. Triangulation was possible because of the multiple interview sources which contributed in developing a robust understanding of the phenomenon of interest.

## Results

**Fig. 1:**
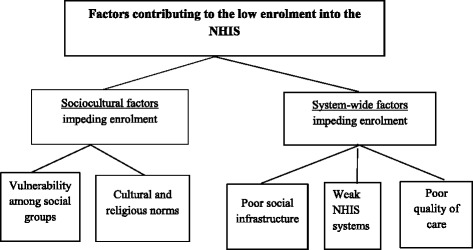
Schematic of results: themes and categories discussed in the text

The qualitative content analysis generated four categories which were developed and are presented here under two broad themes: 1) sociocultural factors and 2) system-wide factors that contribute to low enrolment in the NHIS (see Fig. 1). Some of the example(s) from the dataset were selected that best explain the category. These quotes are presented in italics and between quotation marks.

### Sociocultural factors impeding enrolment

For the scheme to maintain its relevance as a tool to promote universal coverage there is the need to develop the requisite strategies to reduce the barriers that discourage poor and vulnerable groups from accessing healthcare. There are sociocultural factors underlying the process of how and when enrolment in the scheme is sought. We identify two sociocultural factors that contributed to the lack of enrolment in the scheme: 1) Vulnerability among some social groups and 2) Cultural and religious norms.

#### Vulnerability among some social groups

The results show that, issues of respect and support for the aged and vulnerable as well as the social environment in which people access health services exclude them from the NHIS. These groups include the poor, the aged, the disabled, mentally challenged and migrants. Furthermore, although there is a high level of awareness about the exemption scheme among some of the groups such as the aged, the absence of social and family support serves as a barrier to the NHIS.

The following quote describes the vulnerability of the poor during discussions in male and female focus groups:

*“These are the ones we call destitute…There is no way or means where the poor are protected so they benefit from health care… but it is not free for the poor… Rather the government should insure them free of charge, so they enjoy free medical care, because even if they get lucky for someone to pay their health insurance for them, when it expires, they might still not be able to go to the clinic.” (FGD-Previously insured -Nkwantanang)*

*“There is no provision in the NHIS for them. Last week Friday a lady went to the hospital with her sick child but she didn’t have money and she was not having insurance as well. They told her to go find money because without insurance or money they cannot attend to the child.” (FGD-Registered but yet to received ID card-Nkasiem)*.

*“…For an old person like that, when she is sick and you tell her to go to the hospital without anyone, it becomes difficult for her…If anyone is sick and he doesn’t go to the hospital, then that means that the person doesn’t have insurance. And if you don’t have insurance and you go there, it’s a lot of money” (FGG-Insured–Mehame)*.

Vulnerability among some of the social groups stems from several economic reasons and this explains why many people are excluded from the NHIS. The vast majority of people who are excluded from the NHIS are described as the poor because they do not have the financial means to pay the premium for the NHIS. By their very definition, the poor cannot afford the very basic necessities of life and a natural consequence if they are also excluded from the scheme. Transportation costs to the health facilities is a major deterrent even if one had a valid insurance card. A female and male discussants explain this predicament:

*“Because the person has no money, he may not be able to even afford transportation fare. You might have no money and your insurance might have also expired. They might treat you first before they charge you and because your insurance has expired, where will you get the money from. If you are unable to pay, then it means they may have to take back the medication they have given you for you to come back home without being treated” (FGD-Previously insured -Senkyirem).*

*“The insurance is done for free for people beyond a certain age but many of these people can’t even afford the transport fares. In other cases, when the person manages to visit the hospital, they are given medication which they are advised to eat before taking. But because they don’t have money for food they leave the medicine behind…It’s not everyone who can go with the health insurance, because it’s a monetary issue. Today when going for insurance, you ought to carry at least GHC 15.00; this is an amount that a lot of people cannot afford.” (FGD- Insured- Bonwire)*.

#### Cultural and religious norms

Culture is also identified as a major constraint to accessing the NHIS and health care. Some of the women explain that permission must be sought from one’s husband before accessing health care be it curative service or paying the premium for the NHIS, as culture demands that the husband provides the finances needed to access health care. A male from Savelugu describes the influence of religion on women’s enrolment in the scheme:

*“In the Islamic religion, your wife must seek permission before going out… Normally, it is customary that the wife makes the husband aware before going to get the insurance or see the doctor. However, if the husband is not around, and the condition is critical, they cannot go without the knowledge of her husband… because getting the insurance and going to the hospital means spending money. And the money comes from the husband.” (FGD-Never Insured, Savelugu).*

Traditional leaders play a major role in making health services more accessible to their communities and people. They explained that without traditional leadership there is a gap in communication between the government and members of the communities. Communities which do not have strong traditional systems that engage the governmental system lack public services and are therefore cut off from key reform initiatives. Two members allude to this:

*“because there is no chief, there is lawlessness… there is very little or no development facilities such as markets, hospitals etc. because we have no chief in this town…we believe that if there is a chief, it would help us all… if we have a chief in this town, he would stamp his authority, but because there is only a queen mother, the people in the town do not give the requisite respect and act as though there is no authority figure here…the lack of facilities in the town and the distances that we have to travel to reach the ones out of the town. People sometimes die trying to asses them… there are no jobs, thus no money to patronize the health centers” (FGD- Previously Insureds-Abenase)*.

*“the ‘odikiro’[leader] in this community is dead so for me I see that nothing can come…the one to leader us is difficult for us because the queen mother does not stay here the abusuapayin [family head] does not stay here…nobody has time for the community so no one has volunteered to lead for us to get those things done…we sometimes meet and appoint an elder to lead us but when he is called, he does not have the time to go to listen to what they will say” (FGD- Registered but yet to received ID card-Dokyi)*.

The absence of strong leadership undermine the community will within these communities to ensure that basic infrastructure are accessible to members in the community. A general apathy to the needs of the vulnerable has been worsened by apparent lack of leadership to ensure these services are available in the communities.

### System-wide factors impeding enrolment

There are a number of system-wide failures that find their root in the political, historic, and economic structure of communities and are reinforced by local institutions and infrastructure that impact on access to healthcare resources. Consequently, these system-wide factors also impact on the process of enroling in the scheme. Our data reveals three structural factors that contribute to low enrolment in the scheme: 1) poor social infrastructure, 2) weak NHIS systems 3) poor quality of care.

#### Poor social infrastructure

Poor transport and communication infrastructure in many rural communities are noted to exclude many from having adequate access to healthcare. As one traditional ruler stated:

*“the long distance of some villages, as well as a lack of information are the primary reasons why some societies are excluded from getting access to health services”* (traditional ruler).

This is how one scheme manager puts it:

*“We are supposed to register you and give you access by way of giving you a card and a free access to our accredited health facility but if in your community there is no accredited facility and you need to board a car or you need other means to take there that is how the impediment to access will come in”*.

Lack of access to health facilities because of long distances and poor road networks subsequently discourage enrolment in the NHIS. Lack of access to quality information about the NHIS also hinders enrolment. This is how a district social welfare officer observed the situation:

*“If you are aged and you live in the hinterlands how do you know the aged are not supposed to pay for the insurance premium. So if you live in the hinterlands and insurance workers don’t come there you will not be registered or you cannot be registered”* (social welfare officer).

#### Weak NHIS systems

The study shows apparent weaknesses in the key messages sent out, administrative processes within the scheme and systemic problems caused by reforms such as the capitation payment which had been piloted in the Ashanti region at the time of the study. In 2010, the NHIA introduced capitation for payment of primary outpatient services with the aim to contain cost of claims paid to providers while ensuring good quality health services for members of the scheme [18]. Under the capitation system, clients select their preferred health facility, which would be responsible for their primary healthcare needs to simplify the claims process by service providers. Many community members expressed unhappiness with the implementation of the new payment system and had either refused to enrol or had not renewed their expired cards. Consequently, many district schemes had observed declines in their membership rates. A scheme manager states it this way:

*“… but trust me now because of the capitation, our renewal rate has drastically gone down as well as the registration”* (scheme manager).

Some members in the community felt that the initial arrangements with the NHIS was better than the one under the capitation policy. This is because one could just walk into any accredited hospital with little sum of money and still get good care. This is how an opinion leader and male participant in the FGD describes the situation:

*“When the insurance capitation had not come, the attendance was high but when the capitation came, people are no longer attending… because at first the drugs were given in sufficient quality and quantity, but these days the quality and quantity are in question. Some also know the drugs, so when it gets finished they rush to the nearest drugstore”* (opinion leader).

*“Formerly, you could take the health insurance card to any hospital but now because of the capitation you have to go to only one hospital. I am not happy with that so I decided not to renew my membership so I can go to any hospital of my choice when I need help” (FGD, Previously insured-Abenase)*.

Many of the discussions show that most communities do not associate solidarity with the scheme. The NHIS is based on social health insurance principles and one would expect some level of cross-subsidisation between the rich and poor and the healthy and sick. Some of the never-insured claim that they had not enroled in the scheme because they rarely fall sick and are able to use herbal remedies to deal with occasional bouts of illness. Some even describe it as excruciating having an insurance card and renewing it annually when they are not likely to use them or enjoy its benefits, suggesting their lack of awareness of the risk sharing principle underlying the scheme: This how one male participant shares his view:

*“I don’t go to the hospital because I don’t get sick often even. [Even] if I am sick I usually use herbs. It is painful when you don’t use it but have to renew it every year” (FGD, Previously Insured, Abura Amoada)*.

Delays in receiving insurance cards also contribute in the low enrolment rate. Some respondents explain that they had registered with the scheme but had not received their cards which would allow them access to healthcare. According to some, the district schemes had explained that their names and other information they provided had been sent to Accra (capital) for the cards to be prepared and this accounted for the delays. This was a great source of concern to this group of men and women. They concluded that this situation of the insurance scheme is unfair since they had duly paid for what was due them. Three FGD participants captured this concern in two separate group discussions as follows:

*“We registered but the cards are not in. When I was sick yesterday I had to pay. There are even cases where when the card comes you are given an expired card. When you follow them they will tell you that it has been sent to Accra. Some people tend to think it is the fault of the agent. It affects the image of the health insurance agent as people always think that they have spent their monies but if you go to Savelugu yourself, they will tell you the same thing that the card is in Accra” (FGD Registered but yet to received ID card- Langa).*

*“We have done it but still have not received our cards anytime you ask him [the agent] he says it is not ready so when you go you have to pay. That is a problem for us” (FGD, Registered but yet to received ID card.* Denkyira).

*“I have renewed mine for three times but the card never came. I have renewed it for my child for three times and when she takes the picture, it doesn’t show, what can I do about it? I just gave up” (FGD, Registered but yet to received ID card- Dinkyin)*.

#### Poor quality of care

Due to attitudinal and systemic factors, there is a general perception among community members that persons insured with the NHIS do not receive quality healthcare when sick. This perception discourages people from enroling with the NHIS. According to an opinion leader most people in the community prefer to attend the private hospitals because they receive better care there. This is how a community leader and an opinion leader describes the situation:

*“Well, I have complaints of people going to the general and government hospitals and not being attended to, or waiting for so long before they are attended to” (community leader)*.

*“Some of our pregnant women complain that when they go to the hospital they are insulted and because of that, some may be pregnant for about 4 or 5 months without going for their cards and that is very dangerous”* (*opinion leader)*.

One male participants recounts his experience explaining his reason for not renewing his insurance card as stemming from the differential treatment given to NHIS members by health providers:

*“The reason why I have not renewed mine is that, I fell sick and went to the hospital and was told that if I want to be treated, then I have to put the insurance away. And at that time, I saw that they were separating those with the insurance from those without the insurance and they were serving them before us so I changed my mind” (FGD, Registered but yet to received ID card, Abakrampa)*.

But from the perspective of the service providers, the situation is entirely different. They face constraints which undermine the quality of service they could provide to their clients. Delays in the reimbursement of claims by the NHIA greatly impedes their operations. A district medical superintendent explains some of the shortcomings within the NHIS which account for this:

*“We are in 5 months arrears. We have rendered service to clients. We have bought consumables and used them and we are in arrears of 5 months. How do we run the system? And that is the reason why we can run out of stock, and clients and patients will come and for no reason of ours, we do not have the funds and we have to restock, and all you can do is to give them the prescription to go to some recognized pharmacy anywhere to see if they can get their drugs.....you cannot pay the people who have supplied you and clients will lose confidence in the facility and eventually seek other alternatives”* (medical superintendent).

## Discussion

Our study revealed numerous factors contributing to low uptake and renewal of health insurance membership in Ghana. We have categorized these broadly into sociocultural and system-wide factors. Here, based on our findings, we present potential interventions that could help achieve the necessary conditions for all groups of people to participate in the NHIS. These findings as noted in analyzing the discourse between community members, opinion leaders and service providers have produced an understanding of a phenomenon which we find in similar studies in Ghana [19, 20]. We also believe that some of the system-wide factors we identify are not due to the presence of the NHIS but they have historic underpinnings unique to resource allocation shortfalls outside of the health sector and may broadly explain why some communities face difficulties with access to appropriate health care in resource-poor contexts. Some studies have shown that proximity to health providers is a key determinant of choice of care among patients reporting illness [21, 22].

We find that the low uptake in health insurance is framed by two sociocultural factors: 1) vulnerability among some of the social groups particularly the elderly and poor and 2) cultural and religious norms. A show of respect and social support for the aged and vulnerable serve as effective pathways to the inclusion of all persons into the NHIS [23]. Although the findings show a high level of awareness of the exemption policy for the aged, the absence of social and family support serve as a barrier to exclusion from the scheme. In our view, issues of vulnerability raise the dialogue on the economic dimension where the lack of material resources further alienates these social groups from fully participating in the scheme. High transportation costs are a major deterrent to access which explain why communities with health facilities have more people enroling in the scheme [24]. We find that costs associated with travel to health centres and referral hospitals are gravely prohibitive for families in these deprived districts [25, 26]. Also, the fact that members of the scheme are asked by accredited service providers to purchase prescriptions outside of their facilities is an issue of deep concern. Patients complain of high cost of drugs and feel reluctant to renew membership if they still have to face such high charges.

Related to the process of enroling in the scheme, we note the influence of traditional leaders in recommending the scheme to community members and ensuring that necessary logistics are available to enable members to enrol. In the case of weak leadership, where a higher degree of solidarity and the collective voice is missing, members are vulnerable to the incompetence of the scheme and therefore make no effort to enrol or renew their membership in the scheme. In most rural communities, the social practices of community members and individuals are shaped by norms and practices as well as the most dominant religion. As clearly shown in the results, in some communities where the household decisions are taken by the husband or men in the house, this may lead to exclusion of some social groups from accessing the scheme.

We also identify three significant system-wide issues that contribute to low uptake of health insurance: 1) poor social infrastructure 2) weak NHIS system and 3) poor quality of care. Consistent with this framework, our data shows the inequitable distribution of resources along with the underlying issues raised by poverty. Inequities in infrastructure provision is not only a feature in the health sector. Many communities are under served and these inequities worsen the constraints faced by members in accessing health care. However, the inherent weaknesses in the NHIS and its effect on uptake of insurance cannot be overlooked. The NHIS has failed in its key message to community members, whereby people have forgotten that the scheme is built on solidarity and therefore perceive the whole system as one which looks out for individual benefit rather than a collective benefit. The community-based ideology from which the NHIS was built is missing from the responses from community members. Clearly, solidarity and risk-sharing messages have been missing in the media appeal. Related to this perception is the assertion that health insurance is for sick people. This clearly also shows a lack of proper understanding of the concept of insurance.

The NHIS has undertaken some measures to contain its swelling claims costs over the past decade. Claims accounted for 72 % of total NHIS expenditure in 2011 [27–29]. The administrative challenge with the sheer volume of claims which are processed manually and the lack of financial resources to reimburse claims have contributed to the cash shortfalls at accredited health facilities. Health service providers lack the needed funds to procure supplies [30]. Deteriorating service provision signals patients to seek alternate sources of care. Good quality services will stimulate trust in the health system and the scheme which will ultimately influence positively on the uptake and renewal of health insurance and care must be taken to efficiently rollout these cost containment measures [31, 32]. For instance, the backlash from the introduction of capitation as a cost-containment measure sends a clear message that the success of future reforms within the NHIS lies in its educational campaigns within the communities [33]. Effective communication has been identified as crucial in the NHIS uptake and renewal decision; and therefore this is an area which requires improvement to enhance understanding of the scheme and the policy reforms within the scheme.

However, there are some limitations to the study that need to be considered. Both FGDs and individual interviews suffer from some methodological shortcomings but both have distinct features that offer some level of robustness in the results. The FGD method is a limitation due to level of in depth information which is often missing in the results. Yet it is an effective tool for collecting data on opinions, perceptions, values and beliefs [34]. The insight given by individuals with regards to the key challenges to enrolment in the scheme was needed in this study in order to draw out the bottlenecks in the scheme. Key informant interviews allowed us triangulate the data from the FGDs. Yet we are cautious to make any generalisations of qualitative interpretations from the individual interviews and FGDs. Also, the qualitative data obtained in this study was analysed with an inductive approach, as it was considered as the best fit to identify major patterns that explain barriers to enrolment in the scheme. Yet, this might omit some significant themes which were not revealed through the interviews. However, this approach is commonly used when drawing on opinions and perceptions [35] as was the case in the current study.

## Conclusion

The study identifies some major fundamental factors responsible for exclusion from the NHIS as reflected in the discourse of the stakeholders and community members but acknowledges that these are intertwined and can be understood from social, cultural, and system-wide perspectives. The issue of poverty is very important in the decision to enrol in the NHIS. Poor people lack access to economic resources required for better living. Without access to economic resources or because people earn little incomes, it becomes difficult for them to afford enrolment in the NHIS. Although, the NHIS is free for the poor, there has been a huge challenge in identifying who the poor are and there is anecdotal evidence to show that some district officers of the scheme are reluctant to sign up non-paying clients. Also, issues relating to poor health infrastructure often blamed on the unjust and unequal distribution of national resources increases the vulnerability of such disadvantaged groups.

By acknowledging and understanding the key barriers to insurance uptake and renewal, we can begin to explore the value of some of the potential solutions. The NHIS is in the process of introducing key reforms to overcome some of these challenges. The problems associated with the implementation of these reforms may undermine the beneficial effect leaving in its wake misconceptions of what the reforms are meant to achieve in the long run. Interventions should therefore consider the important role of community based structures in applying these measures. Measures that include making health care more affordable by ensuring that communities have good health facilities and skilled personnel and good roads that link them to referral facilities. Communication tools that target communities with messages that are simple and easy to understand will make the NHIS more attractive to people. Therefore, we call for further research to explore what communication processes are needed to inform and educate individuals in relation to enrolment in the scheme in order to expand coverage and ensure sustainability of the scheme.

## Abbreviations

CSOs: Civil Society Organisations
GHS: Ghana Health Service
MOH: Ministry of Health
NGOs: Non-Governmental Organisations
NHIA: National Health Insurance Authority
NHIF: National Health Insurance Fund
NHIL: National Health Insurance Levy
NHIS: National Health Insurance Scheme
NMIMR: Noguchi Memorial Institute for Medical Research
SEND: Social Enterprise Development
SSNIT: Social Security and National Insurance Trust
VAT: Value-Added Tax
WHO: World Health Organization
